# Influence of the composition and shades of ceramics on light transmission and degree of conversion of dual-cured resin cements

**DOI:** 10.1590/1678-7757-2018-0351

**Published:** 2019-07-29

**Authors:** Luana Menezes de MENDONÇA, Ilana Santos RAMALHO, Livia Aguiar Santos Nogueira LIMA, Laís Alcântara PIRES, Thiago Amadei PEGORARO, Luiz Fernando PEGORARO

**Affiliations:** 1 Universidade Tiradentes, Departamento de Prótese, Aracaju, Sergipe, Brasil.; 2 Universidade de São Paulo, Faculdade de Odontologia de Bauru, Departmento de Prótese e Periodontia, Bauru, São Paulo, Brasil.; 3 Universidade Federal do Piauí, Departamento de Prótese, Teresina, Piauí, Brasil.; 4 Universidade do Sagrado Coração, Faculdade de Odontologia, Departamento de Prótese, Bauru, São Paulo, Brasil.

**Keywords:** Ceramics, Polymerization, Resin cements, Fourier Transform Infrared, Dental materials

## Abstract

**Objective:**

Since the transmittance of ceramics can influence the degree of conversion (DC) of resin cements, ceramics composition and shade should be considered in the selection of resin cement. This *in vitro* study aimed to evaluate the effect of the transmittance of different composition, opacities and shades of ceramics on the degree of conversion of two dual-cured resin cements.

**Methodology:**

Sixty discs were prepared from low translucency (LT) and medium opacity (MO) lithium disilicate ceramic, and zirconia ceramic (Z). Each group was subdivided into 5 subgroups (n=4) in shades A2, A3.5, B2, C2 and D3. The transmittance measurement was performed in a spectrophotometer. The Variolink II and Rely X U200 resin cements were photoactivated by LED (1400 mW/cm2) for 40 s through the ceramic discs and without the discs (control group). The DC was measured with infrared FTIR spectroscopy, immediately after light activation. Data were analyzed with Kruskall-Wallis and one-way ANOVA, following post-hoc comparisons by Tukey test and Pearson’s correlation test (P<0.05).

**Results:**

LT ceramic exhibited higher transmittance values compared to MO and Z ceramics. LTA2 and LTB2 showed statistically higher transmittance values compared to MOA2, MOA3.5 and ZA3.5. For Variolink II, the ceramic interposition did not influence the DC, since there were no statistical differences between groups with ceramic interposition and the control group. For Rely X U200 cement, the interposition of some ceramics types/shades (LTA3.5, MOA2, MOA3.5 and ZA3.5) significantly decreased the DC values compared to control group. A positive correlation was found between the ceramic transmittance and DC values of both tested cements. Conclusions. The transmittance and DC values of the cements were influenced by composition and shades of the ceramics. The higher the transmittance of ceramics, the higher the DC values for both cements.

## Introduction

The use of resin cements has grown significantly in recent years due to the increase in demand for ceramic restorations.^1^ The adhesive properties of these cements to the enamel, dentin, metal alloys and ceramics, their biocompatibility and low solubility justify their use, since they promote retention and reduction in the stress concentration at the tooth/cement/restoration interface.^2,3^ So that the physical, mechanical and biological properties of the resin cements are preserved, the light intensity of the light-curing unit must generate sufficient energy to activate and promote an appropriate degree of conversion (DC), since the reduction of the radiation acts negatively in the curing process of these cements.^4-6^ It has been observed that the DC of the monomers in the dual polymerization resin cements is influenced when they are activated under ceramic restorations,^4,6,7^ since these can reduce the radiation reach from the light source to the cement,^8^ and, consequently, alter the properties of the material, which may compromise the success of the ceramic restoration in the medium and long term.

The amount of light that passes through the ceramic depends on its translucency,^5^ which is an optical property that exerts important influence on the aesthetics of the restoration,^9^ and is defined as the property of a material of which most of the transmitted light undergoes scattering, reflecting and transmitting through it. The greater the amount of light through the object, the greater the translucency.^10^ The factors affecting ceramic translucency are numerous, including ceramic composition, crystalline content, thickness,^11-13^ porosity,^14^ and fabrication technique, among others.^3^

The methods for quantifying the transmittance are direct transmission, total transmission (including scattering) and spectral reflectance, and it can be performed in a spectrophotometer.^7^ Among the methods used to evaluate the DC of resinous compounds, the spectrophotometer tests are the most suitable methods for this purpose because they allow detailed measurements of the non-reactive methacrylate groups.^15^

Considering that the transmittance of ceramics can influence the degree of conversion (DC) of resin cements and consequently the longevity of the restoration, the composition and color of ceramics in the selection of resin cement should be considered. The objective of this *in vitro* study was to evaluate the effect of the transmittance of ceramics with different shades and degrees of opacity on the DC of two types of resin cements with dual polymerization modes. The null hypothesis was that ceramics with different degrees of transmittance would not have influence on the DC of the cements.

## Methodology

**Figure 1:**
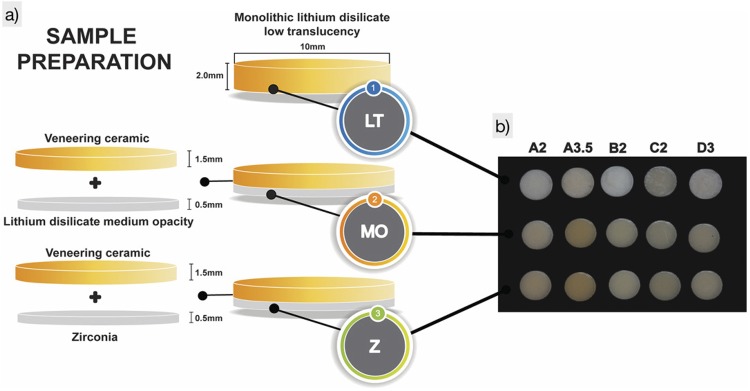
a) Schematic diagram of the specimen preparation; b) Specimens under standardized laboratory light

**Figure 2:**
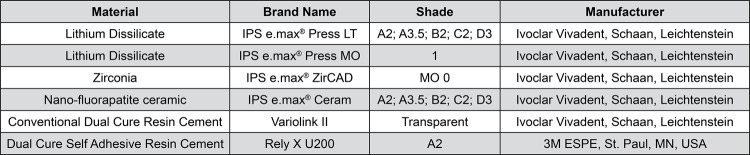
Materials used in this study

Sixty disc-shaped specimens (10.0 x 2.0 mm) were fabricated from lithium disilicate ceramic (IPS e.max^®^ Press, Ivoclar Vivadent AG; Schaan, Liechtenstein) with low translucency (LT) and medium opacity (MO), and zirconia ceramic (Z) (IPS e.max^®^ ZirCAD, Ivoclar Vivadent AG; Schaan, Liechtenstein). Each group was subdivided into 5 subgroups (n=4), according to the ceramic shade: A2; A3.5; B2; C2; D3. In the LT group, the discs simulated monolithic crowns and in the MO and Z groups, the discs were initially made to simulate frameworks and subsequently covered with veneering ceramic (IPS e.max^®^ Ceram, Ivoclar Vivadent AG; Schaan, Liechtenstein) (Figure 1). The descriptions of the ceramics and the resin cements used in this study are summarized in Figure 2.

For lithium disilicate ceramics, disc-shaped wax specimens with 10 mm diameter were initially fabricated in metal matrices with thicknesses of 2.5 mm and 1.0 mm for the LT and MO groups, respectively. The specimens were waxed with casting wax (GEO Classic Natural, Renfert GmbH; Hilzingen, Baden-Württemberg, Germany). The 2.5 mm thick wax patterns were transformed into LT monolithic ceramic discs in the above-mentioned colors, and the 1.0 mm thick wax patterns into MO (color – MO1) ceramic discs. The wax patterns were heat-pressed according to the manufacturer’s instructions with the corresponding shade ingot. For Z group, semi-sintered MO0 zirconia blanks (IPS e.max ZirCAD, Ivoclar Vivadent AG; Schaan, Liechtenstein) were sectioned in a circular shape with a diameter of 12.0 mm (in order to compensate the 20% shrinkage that occurs during sintering) and taken to a cutting machine to obtain 1.0 mm thick slices. After sectioning, the specimens were sintered to full density in a sintering furnace (InFire HTC speed, Dentsply Sirona; Bensheim, Hessen, Germany) at a temperature of 1,500°C for 2 h. All ceramic discs were obtained according to the manufacturer’s specifications. Subsequently, all specimens were ground and polished by using a series of abrasive grinding sheets (#180, 600 and 1200 – Extec, Erios International; São Paulo, São Paulo, Brazil) on an automatic polishing machine (EXAKT 400CS Micro Grinding System, EXAKT Advanced Technologies GmbH; Norderstedt, Schleswig-Holstein, Germany) under constant cooling until uniform thicknesses of 2.0 mm for LT ceramics and 0.5 mm for the MO and Z ceramics had been achieved. The surfaces of the discs were made parallel and polished. The 0.5 mm thick discs (MO and Z) were positioned in a metal matrix (2.5 mm high and 10.0 mm diameter) and the remaining space (2.0 mm thick) was covered with veneering ceramic (IPS e.max^®^ Ceram, Ivoclar Vivadent AG; Schaan, Liechtenstein), following the technique suggested by the manufacturer. The specimens were then ground and polished by using the same abrasive sheet sequence aforementioned until a final thickness of 2.0 mm, with 0.5 mm of the framework ceramic and 1.5 mm of veneering ceramic. The final thickness of each disc was assessed with a digital pachymeter (Mitutoyo Manufacturing Company Ltda; Kawasaki, Kanagawa, Japan). All specimens were immersed in an ultrasonic cleaner with deionized water for 10 minutes and then glazed.

The lithium disilicate ceramic discs received surface treatment with 10% hydrofluoric acid (IPS Ceramic Etching Gel, Ivoclar Vivadent AG; Schaan, Liechtenstein) for 20 seconds, rinsing and air-drying. The zirconia discs were washed and air-dried.

The transmittance was measured by direct transmission method^16^ using a spectrophotometer (UV 1800 spectrophotometer, Shimadzu; Kyoto, Kyoto, Japan). The discs were positioned in a metal template with a limiting window of 8.0 mm diameter to keep it in the optical path of the incident light. The intensity of light transmitted through the specimen was measured continuously at 1 nm intervals, at visible light wavelengths (λ), from 400 to 700 nm. For quantitative analysis, the mean transmittance percentage values were calculated with intervals of 20 nm. The results at a wavelength of 490 nm – corresponding to the maximum absorption of camphorquinone – were considered to determine the transmittance of ceramics influence on the degree of conversion (DC) of the resin cements.

**Figure 3:**
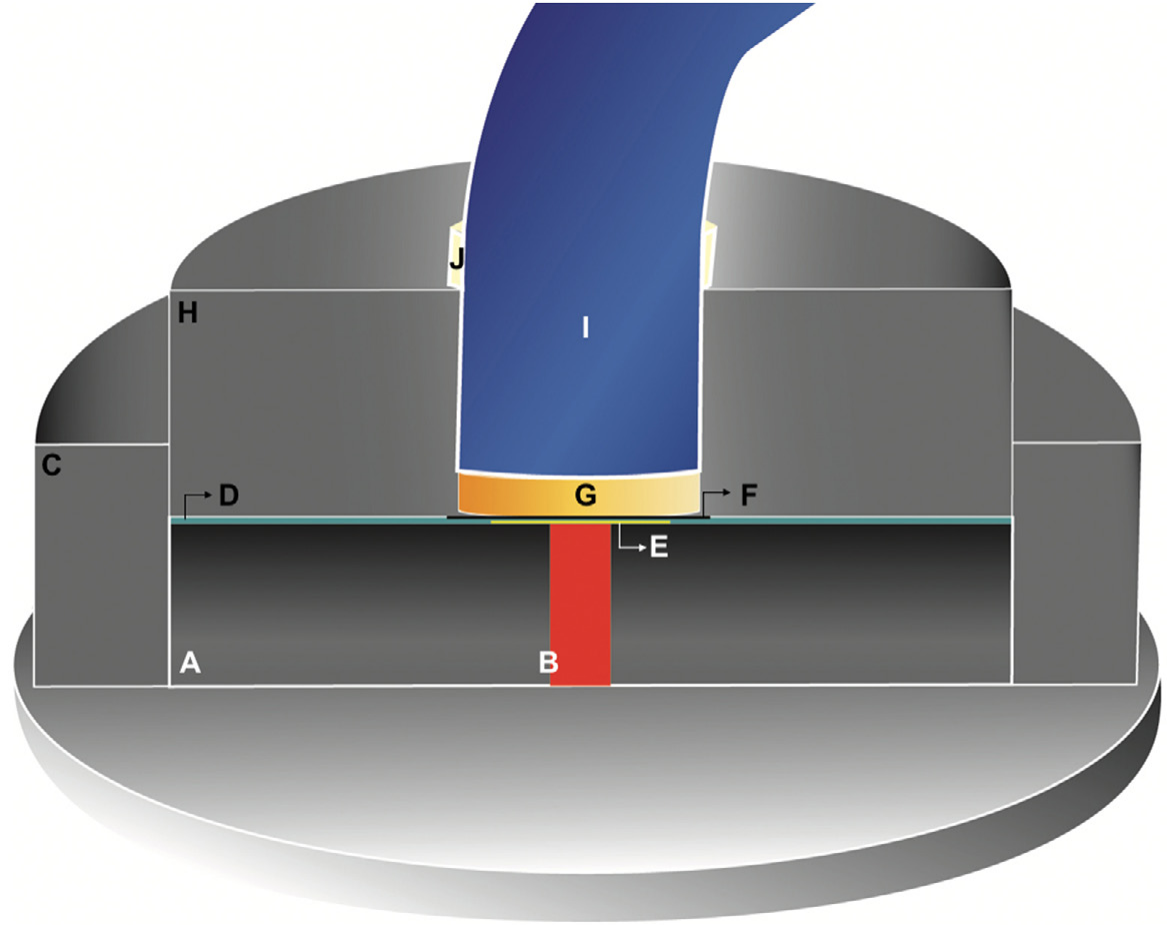
Schematic of the device coupled to the spectrophotometer (longitudinal section). A: Spectrophotometer. B: Spectrophotometer sensor through which the infrared light beam passes. C: Larger ring used to stabilize the spacer strip. D: spacer strip (0.1 mm thick). E: Cement on the diamond surface of the spectrophotometer. F: Polyester strip to prevent direct contact of the cement with discs. G: Ceramic disc. H: Smaller ring that holds the ceramic disc and the tip of the light-curing unit in position. I: Tip of the light-curing unit embedded in the smaller ring and positioned on top of the ceramic disc. J: "Stop" used to standardize the distance of 2.0 mm between the light-curing unit and the cement without the interposition of the ceramic disc

Two dual-cured resin cements, Variolink II (Ivoclar Vivadent AG; Schaan, Liechtenstein) and Rely X U200 (3M ESPE; St. Paul, Minnesota, United States) were used. The degree of conversion (DC) was measured by Fourier transform infrared spectrometer (IRPrestige-2, Shimadzu; Kyoto, Kyoto, Japan) with an attenuated total reflectance (MIRacle™ Single Reflection ATR, Pike Technologies; San Francisco, California, United States) at a resolution of 4 cm^-1^ and 32 scans of each spectrum recorded between the limits 4000 cm^-1^ to 400 cm^-1^. To perform the DC readings, the base and catalyst of both tested cements were provided in equal parts (0.15 g each), weighed on a precision scale and manipulated for 10 s and 20 s for Variolink II and Rely X, respectively, according to the manufacturer’s specifications. To standardize the cement thickness (100 μm) and the distance and position of the curing light (2.0 mm) to the spectrophotometer, a metallic device was coupled to the ATR, as described in Figure 3. Initially, the DC of the unpolymerized cement was performed. The absorption peaks of the aromatic double bonds were recorded at 1608 cm^-1^ and the peak of the aliphatic double bonds (C=C) were registered at 1636 cm^-1^. Subsequently, the ceramic disc was placed under finger pressure on the cement with the interposition of a polyester strip to prevent direct contact of the cement with discs. Then, the tip of the photopolymerizer (DB 685, Dabi Atlante; Ribeirão Preto, São Paulo, Brazil) was embedded in the metallic device and supported on the top of the ceramic disc. Both cements were light-cured for 40 seconds with a LED curing unit (DB 685, Dabi Atlante; Ribeirão Preto, São Paulo, Brazil), with an irradiance of 1,400 mW/cm^2^, which was regularly determined by a radiometer (Model 100, KERR Corporation; Orange, California, United States). The FTIR readings were obtained immediately after light activation. For each composition/shade of ceramics and cements, 5 readings were performed. In addition, five more readings were performed in the cured cements without an interposition of the ceramic discs, serving as a control. In this case, a polyester strip was interposed between the resin cement layer and the light-curing unit tip and a “Stop” was used to standardize the distance of 2.0 mm between the light-curing unit and the cement. The determination of DC was performed according to the following equation: DC%=100x [1- (R_cured_/R_uncured_)], where R represents the ratio between 1638 cm^-1^/1608 cm^-1^ of polymerized and unpolymerized cement.^17^ The transmittance and DC evaluations were performed by one examiner.

Statistical evaluation of transmittance was performed using the nonparametric Kruskal-Wallis test and the Tukey *post-hoc* test for multiple comparisons between groups. Regarding the DC evaluation, the Kolmogorov-Smirnov statistical test for normality and Levene’s test for homogeneity revealed normal distribution for data (*P*>0.449 and *P>0.*802, respectively). Due to the different composition, the DC values of each cement were evaluated separately. Therefore, DC values were analyzed using one-way analysis of variance (ANOVA) and the Tukey *post-hoc* test for multiple comparisons between groups to evaluate the effect of the ceramic interposition (Control, LT, MO and Z) and the ceramic shade (A2, A3.5, B2, C2 and D3) on the DC. Pearson’s correlation test was used to evaluate the influence of transmittance on the DC of the cements. The significance level was set at *P*=0.05. All analyses were accomplished using the software Statistic for Windows, version 13 (Sigma Plot v12.0, Jandel Scientific; San Jose, California, United States).

## Results

**Figure 4:**
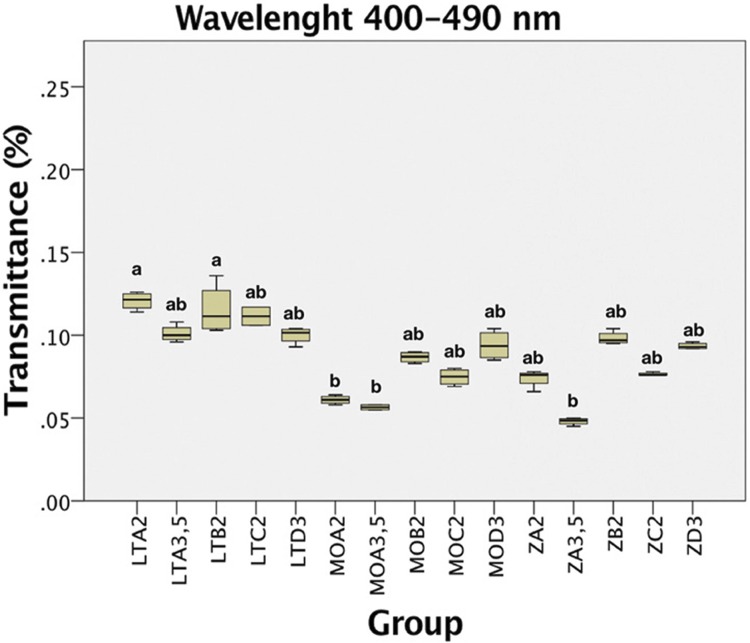
Box plot of transmittance (%) at 400-490 nm wavelength interval as a function of ceramic composition and ceramic shade. *Identical letters indicate statistically homogeneous groups

The transmittance (%) mean values of the different ceramics and shades at the wavelengths of 490 nm are shown in Figure 4. Kruskal-Wallis test revealed that the ceramics composition and shades affected the transmittance (*P<0*.01). In general, the LT ceramic exhibited higher transmittance values (0.11%) compared to MO (0.075%) and Z (0.078%) ceramics. Results from the pairwise comparisons by Tukey test for each group depicted that LT A2 and LT B2 showed statistically higher transmittance values compared to MO A2, MO A3.5 and Z A3.5 (all, *P*<0.01*).* All other group comparisons were not significantly different (*P>0.*05).

**Figure 5:**
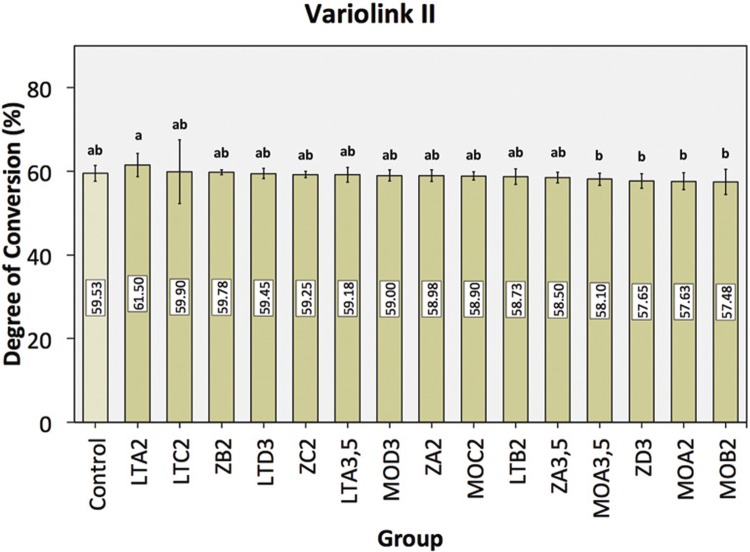
Mean DC percentage ± confidence interval of Variolink II as a function of ceramic composition and ceramic shade. *Identical letters indicate statistically homogeneous groups

Concerning the results from DC evaluation, the cements were analyzed separately due to their different compositions. The DC mean values with the corresponding 95% confidence interval (CI) of the Variolink II, without (control group) and with the superposition of the ceramic discs in the different shades, are shown in Figure 5. Results from one-way ANOVA revealed that the ceramic superposition did not influence the DC, since there were no statistical differences between groups with ceramic interposition and the control group (*P*>0.321). Additionally, the pairwise comparison for each group showed that LT A2 ceramic presented statistically higher DC values compared to MO ceramic in shade A2 (*P*=0.001), A3.5 (*P*=0.004), and B2 (*P=0*.001) and Z ceramic in shade D3 (*P=*0.003).

**Figure 6:**
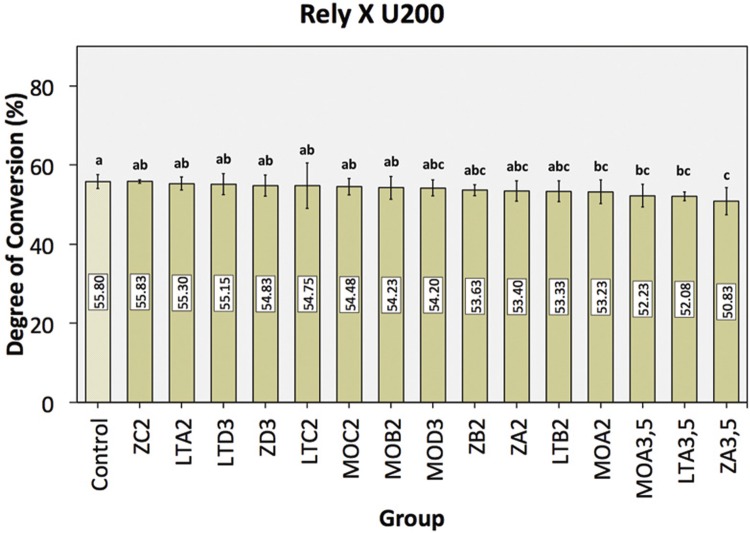
Mean DC percentage ± confidence interval of Rely X U200 as a function of ceramic composition and ceramic shade. *Identical letters indicate statistically homogeneous groups

The DC mean values with the corresponding 95% confidence interval (CI) of the Rely X U200 are shown in Figure 6. Results from one-way ANOVA revealed that the composition and shade of ceramics affected the DC of the Rely X U200 (*P*=0.000; F=4.46). When comparing the DC values as a function of both factors, the pairwise comparison for each group revealed that the control group exhibited statistically higher DC values compared to groups with the superposition of the LT ceramic in shade A3.5 (*P=0.*007*)*, MO ceramic in shades A2 (*P=*0.047*)*, A3.5 (*P=*0.010*)* and Z ceramic in shade A3.5 (*P=*0.000*)*.

The Pearson correlation coefficient value illustrated that there was a weak positive correlation between the ceramic transmittance and DC values from Variolink II and Rely X U200 (r^2^= 0.421, P=0.001 and r^2^= 0.330, P=0.011, respectively), which means that the higher the transmittance of ceramics, the higher the degree of conversion of cements.

## Discussion

This study investigated the influence of the transmittance of different ceramic composition and shades on the degree of conversion (DC) of two dual-cured resin cements. The null hypothesis was rejected because the transmittance values varied in the different ceramic types and shades and affected the DC of the cements. There was a positive correlation between the ceramic transmittance and DC values of both tested cements.

One of the paramount success factors of ceramic restorations consists on a strong and stable bond between the ceramic and luting agent and, respectively, between cement and dental structures.^4^ In case of the use of resin cements as a luting material, the bond strength will be determined by reaching satisfactory resin polymerization.^4-6^ Nonetheless, in the polymerization through ceramics, a significant quantity of light will be lost by absorption, scattering or transmission, which may impair the final polymerization of either a light-cured or dual-cured resin cement, and, consequently, the longevity of restoration.^4,7^

The light energy passing through a translucent material can be quantifying by three methods: direct transmission, total transmission, and spectral reflectance.^16^ The method used in this study was the direct transmission. Therefore, the values of translucency obtained through the spectrophotometer corresponded to the amount of light (direct beams, without quantifying the scattered light) that reached the detector of the spectrometer. It is characteristic of this methodology to obtain values lower than 1% for dental ceramics. In this study, the transmittance of ceramics in the visible light wavelength (400 to 700 nm) corresponded to at maximum 0.23%, with an average of 0.18% for all tested ceramics. Broadbelt, et al.^16^ (1980) have found similar results, averaging 0.13% of light that passed directly on feldspathic ceramic specimens of 1.0-mm-thickness, using the same wavelength interval. Considering that in the light-curing process of resin cements, is the most commonly used photoinitiator in such materials, presenting an absorption peak at 470 nm,^4^ corresponding to the blue light spectrum, the results of a narrow wavelength (400-490 nm) were considered to determine the influence of transmittance on the resin cements DC.

Regarding the transmittance analysis of the different ceramics and shades at the wavelengths of 490 nm, the results of this study revealed that the composition and shades of ceramics affected the transmittance (*P<0*.01). In general, the LT ceramic exhibited higher transmittance values (0.11%) compared to MO (0.075%) and Z (0.078%) ceramics. Even though, LT and MO are disilicate-based ceramics, the significant differences between them may be explained by the differences in opacity and preparation technique of the specimens, since the latter had a form of framework covered with a veneering ceramic by the stratification technique. It has already been observed that the application of a veneering ceramic decreases light transmission because of the high volume of porosity and reflectance that occurs at the interface between the framework and the veneering ceramic.^14^ Based on these findings, it is noteworthy that, zirconia ceramic, even having a higher degree of opacity, may be considered translucent to a certain extent, since such ceramic showed similar transmittance values as those of the MO group, which in theory is more translucent due to the smaller amount of crystalline structure characteristic of vitreous ceramics.^9,10,14,18^ Concerning the pairwise comparison for each group as a function of both factors, LT A2 and LT B2 showed statistically higher transmittance values compared to MO A2, MO A3.5 and Z A3.5. In general, it can be inferred that the preparation technique along with the saturation (chroma) of the ceramics influenced the transmittance values, more than shade (hue) and composition. Previous investigations, demonstrated that the transmittance is influenced negatively by the degree of opacity, thickness and shade saturation of the ceramics.^19-21^ However, the comparison of results between different studies is jeopardized by differences in methodologies used, as well as the different composition and shades of ceramics.^9,10,18,19,22,23^

Among the several methodologies to determine the polymerization quality of composites, the spectrometry has been widely employed in various studies.^6,12,24-33^ This research showed that the two cements behaved differently when light-activated under the same conditions, although both were dual cure resin cements. The polymerization reaction of the cements is influenced by the composition, such as monomer type and inorganic particle content.^24,29^ Since the DC is dependent on the composition of the cements, comparisons between cements with different compositions should be avoided. Therefore, the DC values of each cement were evaluated separately.

The DC results of this work showed that the composition and shades of the ceramics had an influence on the DC of Variolink II and Rely X U200 cements. For Variolink II, it was found that LT A2 ceramic presented statistically higher DC values compared to MO ceramic in shade A2, A3.5, and B2 and Z ceramic in shade D3. Nonetheless, despite some statistical differences within ceramic groups, the most important finding was that the interposition of ceramic disc did not influence the DC of this cement, since there were no significant differences between groups without (control group) and with interposition of ceramic discs in different shades, which is a reassuring result for the clinician. This fact may be attributed to the amount of photoinitiators and sensitivity of the cement when light-cured even under ceramic discs. As explained by Ilie and Hickel^4^ (2008), the velocity of the photo-initiation polymerization reaction is limited and an unrestricted increase in irradiation will not be able to accelerate this process. Furthermore, in a light activated polymerization system, the effectiveness of an initiator is limited by a deactivation mechanism. Not only can an active photoinitiator start a polymerization reaction but, by recombination with another active photoinitiator or reaction with an activated polymer chain, it can also stop it. Therefore, the amount of light that reached the cement layer underneath the ceramic was enough to achieve a degree of polymerization equivalent to the control group without ceramic interposition. Although some authors obtained similar results between specimens without and with the superposition of ceramics disc,^34^ different results were found by other investigations.^6,19^

The Rely X U200 cement presented significant differences between the groups without and with disc superposition for the LT ceramic in shade A3.5, for the MO in shades A2 and A3.5, and for the Z ceramic in shade A3.5. Other studies also found differences with the Rely U100 cement when light-activated without (56.7%) and under IPS Empress 2 (49.7%) with 2.0 mm thickness.^31^ Flury, et al.^35^ (2013) showed that discs of IPS Empress CAD and IPS e.max CAD with a thickness of 1.5 mm did not significantly influence the DC of the Rely X Unicem 2 cement (50.2% and 49.5%, respectively) when compared to the DC of the polymerized cement without the superposition of discs (51.1%). It is observed that the above-mentioned studies used cements with different trademarks, but basically with the same composition.

When correlating the transmittance and DC results, the Pearson’s correlation test showed a significant weak positive correlation between these two aspects for both cements, showing that the higher translucency value, the higher was the DC of the cements. The results of this study are in agreement with previous scientific evidence,^4,19,20,25,26,36,37^ which stated that differences in shade and opacity of restorative materials influence transmittance and, consequently, the polymerization of the cements. It seems reasonable to emphasize that monomer conversion of resin cements is closely related to the amount of total light transmitted (direct and scattering light) through the indirect restoration. Nonetheless, in the current study the values of transmittance were obtained using the direct transmission method (direct beams, without quantifying scattering light, as previously mentioned). Accordingly, this fact could explain the lower correlation found in comparison to other authors.^4,20,25,36^

In general, the DC values of the cements under the conditions tested in this work were similar as a function of the 2.0-mm-thickness of the discs. This aspect is corroborated by other studies^4,11,28,33,36-38^ that did not show differences in hardness and DC values when the ceramic presented thickness up to 2.0 mm. Despite the significant differences between the DC of the resin cements under different ceramic composition and shade, its important to highlight that the DC values of the two cements were between 50 and 70%, with a slight difference concerning the control group (approximately 5%). As demonstrated in previous investigations,^6,39^ it seems reasonable to emphasize that such DC values are sufficient for resin cements to have an acceptable clinical performance. It also is important to highlight that the DC measurements were performed immediately after light activation. It is expected that the DC gradually expands as a function of time, with a substantial increase in the first 30 min and with a linear increase up to the first 24 h.^8,24,40^ Therefore, according to the results, it can be stated that the cements used in this study may be used with the ceramics tested without causing relevant clinical impact in the DC of these cements.

## Conclusions

Within the limitations of this *in vitro* study, the following conclusions were made:

There was a significant difference in the values of transmittance between some composition and shades of ceramics. The LT ceramic presented the highest transmittance values, followed by the MO and Z ceramics.

The superposition of the ceramic discs had no influence on the DC of Variolink II cement. For Rely X U200, the ceramic disc negatively influenced the DC.

There was a positive correlation between transmittance of ceramics and DC of Rely X U200 and Variolink II.
